# Association of Inflammasome Gene Expression Levels with Pathogenesis of Familial Mediterranean Fever in Armenians

**DOI:** 10.3390/ijms252312958

**Published:** 2024-12-02

**Authors:** Varduhi Hayrapetyan, Lana Karapetyan, Lilit Ghukasyan, Sofi Atshemyan, Hovsep Ghazaryan, Valentina Vardanyan, Vahan Mukuchyan, Arsen Arakelyan, Roksana Zakharyan

**Affiliations:** 1 Institute of Biomedicine and Pharmacy, Russian-Armenian University, Yerevan 0051, Armenia; varduhi.hayrapetyan@rau.am (V.H.); h_ghazaryan@mb.sci.am (H.G.); 2 Laboratory of Human Genomics, Institute of Molecular Biology of the National Academy of Sciences of the Republic of Armenia (NAS RA), Yerevan 0014, Armenia; l_ghukasyan@mb.sci.am; 3 Department of Rheumatology, Yerevan State Medical University after Mkhitar Heratsi (YSMU), Yerevan 0025, Armenia; valentina.vardanyan@gmail.com; 4 Department of Rheumatology, “Mikaelyan” Institute of Surgery, Yerevan 0052, Armenia; 5 Nairi Medical Center, Yerevan 0015, Armenia; vmukuchyan@gmail.com

**Keywords:** Familial Mediterranean fever, inflammasome, immunity, gene expression, MEFV gene

## Abstract

Familial Mediterranean fever (FMF) is a genetically determined autoinflammatory disease transmitted mostly by an autosomal recessive mechanism and caused by point mutations of the *MEFV* (Mediterranean FeVer) gene. The aim of this study was to evaluate the expression of inflammasome genes (*p65*, *Casp1*, *MEFV*, and *NLRP3*) in patients with FMF compared to controls to understand the changes playing a key role in disease development. We found altered expression levels of the full-length *MEFV* isoform as well as *Casp1* and *p65* in FMF patients versus controls. This, once again, highlighted the significance of inflammasome genes in terms of FMF.

## 1. Introduction

Familial Mediterranean fever (FMF) is a genetically determined autoinflammatory disease with a mostly autosomal recessive inheritance pattern. The cause of the nosology are point mutations of the *MEFV* (Mediterranean fever) gene coding pyrin protein, also known as marenostrum [1,2,3,4].

Pyrin is mainly expressed in the cells of the innate immune system, namely, granulocytes, eosinophils, monocytes, dendritic cells, and synovial fibroblasts [2]. It is composed of five domains, namely, PYRIN (1–92), bZIP (266–280), B-box zinc finger (370–412), a-helical (420–440), and B30.2 (597–776), responsible for various protein–protein interactions [2]. Several alternatively spliced transcripts of pyrin encoding the *MEFV* gene have been identified [5,6]. The first described alternatively spliced isoform, lacking exon 2, *MEFV*-d2, is found at a higher ratio in mononuclear cells compared to the full-length *MEFV* isoform [7,8,9,10].

Pyrin is a key component of the pyrin inflammasome which is a molecular platform responsible for the activation of caspase-1 (Casp-1), a cysteine protease mediating proteolytic processing and the activation of the proinflammatory cytokines IL-1β and IL-18 [11,12,13]. Usually, inflammasomes are formed as a result of the oligomerization of a nucleotide-binding domain-like receptor (NLR), such as NLRP1, NLRP3, NLRC4, and AIM2 proteins [10]. Inflammasome dysfunction results in the activation of inflammatory cascades and the hyperactivation of innate immunity [11], which are the main hallmarks of FMF.

The aim of this study was to evaluate the expression of inflammasome genes (*p65*, *Casp1*, *MEFV*-d2, *MEFV*-I, and *NLRP3*) in patients with FMF compared to healthy subjects (a reference control group) to understand the changes that may play a key role in disease development. These findings might complement the existing data on functional changes in inflammasome genes in FMF and underline the major alterations in immunity present in patients with FMF. The results obtained might find further use in the development of more accurate diagnostic and prognostic approaches for this disease. 

## 2. Results

This study was aimed to evaluate the expression of inflammasome genes in patients with FMF compared to healthy subjects (a reference control group) to underline the changes implicated in disease pathogenesis. 

In total, 29 FMF patients (male/female: 10/19) and 20 healthy controls (male/female: 7/13) participated in inflammasome gene expression analysis using quantitative real-time polymerase chain reaction (qRT-PCR).

**Figure 1:**
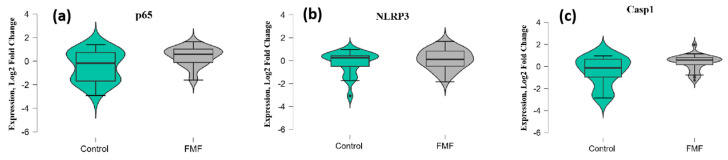
mRNA expression levels of *p65* (**a**), *NLRP3* (**b**), and *Casp1* (**c**) in patients with FMF and healthy controls. The horizontal line that splits the box in two represents the median; the lower and upper sides of the box represent the 1st (Q1) and 3rd (Q3) quartiles; and whiskers represent the 1.5 interquartile range from Q1 and Q3.

The mean mRNA expression levels for *p65*, *NLRP3*, *Casp1*, and *MEFV* isoforms were calculated as log_2_ fold change. The mean expression levels for *p65* were significantly higher in FMF patients than in controls (patients vs. controls, mean ± SD, −0.45 ± 1.29 vs. 0.42 ± 0.79; *p* = 0.02) (Figure 1a). Concerning *NLRP3*, there was no significant difference in expression between the patients and controls (patients vs. controls, mean ± SD, −0.18 ± 1.00 vs. 0.006 ± 1.16; *p* = 0.33) (Figure 1b). In contrast, the difference in the expression of *Casp1* reached statistical significance between the groups (patients vs. controls, mean ± SD, −0.44 ± 1.31 vs. 0.39 ± 0.71; *p* = 0.048) (Figure 1c).

**Figure 2:**
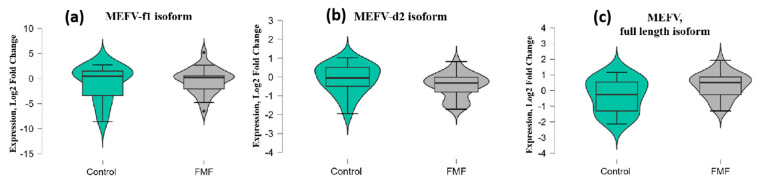
mRNA expression levels of *MEFV*-f1 (**a**), *MEFV*-d2 (**b**), and *MEFV* full-length (**c**) isoforms in patients with FMF and healthy controls. The horizontal lines that splits the box in two represents the median; the lower and upper sides of the box represent the 1st (Q1) and 3rd (Q3) quartiles; and whiskers represent the 1.5 interquartile range from Q1 and Q3.

Among three isoforms, only the expression of the *MEFV* full-length (i1.2) transcript was borderline significant (patients vs. controls, mean ± SD, −0.34 ± 1.1 vs. 0.32 ± 0.79; *p* = 0.049) while for the transcript lacking exon 2 (*MEFV*-d2), *p*-values exceeded the significance threshold (patients vs. controls, mean ± SD, −0.1 ± 0.79 vs. −0.45 ± 0.65; *p* = 0.08) (Figure 2a–c). 

Thus, our study indicated changes in the expression of the genes *Casp1*, *MEFV*, and *p65* in patients with FMF compared to healthy individuals, reinforcing earlier findings regarding the activation of the pyrin inflammasome in autoinflammatory conditions like FMF, and the absence of notable differences in *NLRP3* expression between the two groups may indicate that the pyrin inflammasome plays a more prominent role than the NLRP3 inflammasome in this context.

## 3. Discussion

In this study, we aimed to evaluate the levels of *MEFV* and other inflammasome genes in FMF in comparison with healthy subjects. Previous studies indicated that in monocytes, the expression of *MEFV* is variable and upregulated by proinflammatory cytokines, interferon-γ (IFN-γ), tumor necrosis factor-α (TNF-α), lipopolysaccharide (LPS), and interleukin 1 (IL-1) [9]. It has been shown that *MEFV* gene mutations might lead to changes in pyrin activity, altering various reactions of innate and adaptive immunity [10]. Thus, several lines of evidence also suggested an impact on inflammasome activity [11]. 

Being a part of the pyrin inflammasome, pyrin acts as a pattern recognition receptor, sensing pathogen modification and the inactivation of Rho GTPases, and also by directing the inflammasome components NLRP1, NLRP3, and pro-caspase 1. Pyrin modulates the activation of Casp-1 and IL-1β, resulting in proinflammatory or anti-inflammatory reactions [10]. Several studies indicated the implication of both the pyrin and NALP3 inflammasomes in the pathogenesis of autoinflammatory diseases including FMF [7,10,11,14]. Previously decreased levels of NLRP3 and Casp1 in whole blood cells extracted from FMF patients compared to controls have been detected. Subsequent treatment with LPS resulted in reduced IL-1β production mediated by decreased levels of NLRP3 among the patients [14]. Several studies, including a report by Grandemange et al. (2009), hypothesized that insufficient *MEFV* expression might play a crucial role in the physiopathological conditions underlying FMF [4]. 

In physiologic conditions, RhoA GTPase activates the serine–threonine kinases PKN1 and PKN2 that bind and phosphorylate pyrin [10]. Phosphorylated pyrin binds to inhibitory 14-3-3 proteins, and this process maintains pyrin in an inactive state that prevents the formation of the pyrin inflammasome. In FMF, mutations in the *MEFV* gene impair the interaction of pyrin with microtubules, PKN, and 14-3-3 proteins, facilitating the formation of a proinflammatory pyrin inflammasome. When the pyrin inflammasome is assembled, it activates caspase-1 to process pro-IL-1β and pro-IL-18 to their mature forms IL-1β and IL-18, respectively, and cells undergo an inflammatory death termed pyroptosis [11]. The genes involved in the formation of the pyrin inflammasome include *NLRP3*, *Casp1*, *GAPDH*, and *p65*. The excessive activation of the pyrin inflammasome leads to the inflammation that characterizes the febrile inflammatory episodes seen in FMF [11].

Repa et al. found that the LPS/ATP activation of NLRP3 resulted in lower levels of IL-1β in FMF patients compared to healthy individuals. Similarly, the overexpression of the M694V-mutated pyrin in THP-1 cells decreased the release of IL-1β mediated by NLRP3 [14]. Previous findings by Chae et al. (2006) revealed the tendency of increased levels of Casp1 in three common FMF-associated B30.2 mutations [15]. It must be noted that mutations in pyrin also result in the heightened activation of the NF-κB (P65) gene [16,17]. However, the way *MEFV* mutations in FMF patients affect inflammasome activity is still unclear and needs further investigation. Furthermore, the specific impact of *MEFV* sequence variations on the pyrin inflammasome response remains largely unexplored. Jamilloux et al. (2018) reported that FMF severity is tightly linked to the nature of the *MEFV* mutation and to the number of mutated alleles [18].

A few limitations related to our study must be considered. A limitation of our study is the relatively small sample size in each group; therefore, the results obtained can be considered as preliminary. Moreover, because of the first limitation, we did not explore the potential relationship between the presence of pathogenic variants of *MEFV* and inflammasome gene expression levels; we plan to do that in the near future. Moreover, it is planned to evaluate the inflammasome expression levels in FMF patient-specific induced pluripotent stem cell lines depending on the presence of genetic variants.

## 4. Materials and Methods

### 4.1. Study Population and Selection Criteria

In total, 29 patients with FMF and 20 healthy subjects were enrolled. Mutations in the *MEFV* gene in the FMF patients was determined by commercially available qPCR assay for the 26 most common mutations with an FMF Multiplex real-time PCR kit (SNP Biotechnology RnD Ltd., Ankara, Turkey). This qPCR kit determines 20 mutations, which have been identified in exon 1 (E84K), in exon 2 (L110P, E148Q, E148V, E167D, E230K/Q, T267I, P283L, and G304R), in exon 3 (P369S), in exon 5 (F479L), and in exon 10 (M680I (G/C-A), M694I, M694V, K695R, V726A, A744S, and R761H), covering 99.2% of the mutations of FMF in Anatolian, Middle Eastern, and many other countries. Patients with at least 1 functional mutation in the *MEFV* gene and the appearance of clinical symptoms of FMF were enrolled in this study.

All subjects gave their informed consent to participate in the study, which was approved by the Ethical Committee of the Institute of Molecular Biology of the National Academy of Sciences RA (IRB #00004079, Protocol N3 from 23 August 2021). 

### 4.2. RNA Extraction and cDNA Synthesis

Five ml of morning fasting blood was collected in EDTA-containing tubes. The extraction was performed using a Quick-DNA/RNA Miniprep Plus Kit (Zymo Research, Irvine, CA, USA), according to the manufacturer’s instructions. For all extracted RNA samples, measurements of concentrations and purity were performed using a nano spectrophotometer (DeNovix DS-11+, USA) and a Qubit 4 Fluorometer with Qubit RNA HS Assay Kit (ThermoFisher Scientific, Waltham, MA, USA). Only highly purified samples with an A260/A230 ratio within the 1.7–2.2 range and a concentration within the 10–47 ng/μL range were used. The extracted RNA samples were stored at −80 °C until further use at the Center of Excellence for Genome Editing and 3rd Generation Sequencing at the Russian-Armenian University (Yerevan, Armenia). 

cDNA synthesis was performed by reverse transcription (8 μL total RNA; total volume of cDNA 10 μL) using a LunaScriptTM RT SuperMix Kit (New England Biolabs, Ipswich, MA, USA), with primer annealing for 2 min at 25 °C, cDNA synthesis for 10 min at 55 °C, and heat inactivation for 1 min at 95 °C. The cDNA samples were stored at −20 °C until further use.

### 4.3. Evaluation of Inflammasome Gene Expression

To assess the expression of inflammasome genes, primers targeting the genes *MEFV*, *p65*, *Casp1*, *NF-kB, NLRP3*, and *GAPDH* as a housekeeping gene were used. They were measured by quantitative real-time polymerase chain reaction (qRT-PCR) on the Rotor-Gene™ Q machine (Qiagen, Hilden, Germany).

Three μL of cDNA was added to 20 μL of PCR-mix. The final reaction mix contained 4 μL of 5x HOT FIREPol^®^ EvaGreen^®^ qPCR Mix Plus (Solis BioDyne, Estonia; content: HOT FIREPol^®^ DNA Polymerase, 5x EvaGreen^®^ qPCR buffer, 12.5 mM MgCl_2_ (1x PCR solution—2.5 mM MgCl_2_), dNTPs, EvaGreen^®^ dye), 0.5 μL of reverse and forward primers (for the housekeeping gene, 0.375 μL of both forward and reverse primers was used), and nuclease-free water to adjust the final volume to 20 μL.

qPCR was performed using the following thermal cycling conditions: initial activation for 12 min at 95 °C, 40 cycles of denaturation for 15 s at 95 °C, annealing for 30 s at 60 °C, and extension (acquiring on green) for 30 s at 72 °C.

The primers and fluorescently labeled probes were selected using the PrimerQuest tool (Integrated DNA Technologies), as described in Table 1. 

### 4.4. Statistical Analysis

The distribution of gene expression data was checked for normality using the Shapiro–Wilk normality test. Since our data did not conform with normality assumption, we performed independent sample pairwise comparisons using the non-parametric Mann–Whitney U test. *p* values < 0.05 were considered significant. Statistical calculations were performed using JASP software (version 0.18.1). The delta-delta Ct method (also known as the 2^−∆∆Ct^ method) was used to calculate the relative changes in inflammasome gene expression from quantitative real-time PCR experiments [19]. Data were presented as log2 fold change values. The average Ct values of control groups were used as a reference in Ct calculations. 

## 5. Conclusions

In conclusion, our study revealed the altered gene expression of *Casp1*, *MEFV*, and *p65* in patients with FMF compared to healthy subjects, supporting the previous findings on the activation of the pyrin inflammasome in autoinflammatory diseases including FMF. The lack of significant differences in *NLRP3* expression between the comparison groups might reflect the more significant contribution of the pyrin inflammasome than the *NLRP3* inflammasome in our terms. However, additional studies with larger sample sizes are needed to clarify this issue.

## Figures and Tables

**Table 1:** QRT-PCR probes/primers for inflammasome genes used in this study.

1	GAPDH forward	AGCCTCAAGATCATCAGCAAT
	GAPDH reverse	GTCATGAGTCCTTCCACGATAC
2	P65 forward	CTGTCCTTTCTCATCCCATCTT
	P65 reverse	ACACCTCAATGTCCTCTTTCTG
4	Casp1 forward	TTTCCGCAAGGTTCGATTT
	Casp1 reverse	CCTGGGAAGAGGTAGAAACA
5	MEFV i1 forward	ATTCGGTCACCGGAAGG
	MEFV i1 reverse	GGAATCACGCACACAGGTA
6	MEFV i2 forward	ACTCTGCTGGTCACCTACTAT
	MEFV i2 reverse	GGTGGCCTTCCCTGAATG
7	MEFV i2 forward	GGCAGCCATTCAGGGAAG
	MEFV i2 reverse	GGAATCACGCACACAGGTA
8	MEFV i1.2 (full-length) forward	AGATCAGGAAGGCATATGACAC
	MEFV i1.2 (full-length) reverse	CTCCAATGTCCTGCAGAAGT
9	NLRP3 forward	GAAGAGGAGTGGATGGGTTTAC
	NLRP3 reverse	CTTTCTGTACTTCTTACGGTAATCTTTC

## Data Availability

The data presented in this study are available upon request (Data are contained within the article and Supplementary Materials).

## Supplementary Materials

The following supporting information can be downloaded at: https://www.mdpi.com/article/10.3390/ijms252312958/s1.
