# Supplementary material for: Association of Inflammasome Gene Expression Levels with Pathogenesis of Familial Mediterranean Fever in Armenians
Source: Int J Mol Sci. 2024 Dec 2;25(23):12958. doi: 10.3390/ijms252312958 (PMC11641286; doi:10.3390/ijms252312958)

Results

Independent Samples T-Test

Independent Samples T-Test

	Test	Statistic	df	p
p65_s1	Student	−1.197	42.000	0.238
	Welch	−1.193	38.399	0.240
	Mann-Whitney	197.000		0.347
p65_s3	Student	−2.954	47.000	0.005
	Welch	−2.710	28.726	0.011
	Mann-Whitney	176.000		0.020
casp_s1	Student	−2.844	47.000	0.007
	Welch	−2.564	26.644	0.016
	Mann-Whitney	192.500		0.048
mefv_i1_s1	Student	−0.446	46.000	0.658
	Welch	−0.424	32.851	0.675
	Mann-Whitney	291.000		0.828
mefv_i2_s1	Student	1.647	44.000	0.107
	Welch	1.593	34.022	0.120
	Mann-Whitney	334.000		0.086
mefv_i2_s2	Student	1.476	42.000	0.147
	Welch	1.385	24.912	0.178
	Mann-Whitney	296.000		0.191
mefv_i1.2_s2	Student	−2.410	45.000	0.020
	Welch	−2.248	28.560	0.033
	Mann-Whitney	170.500		0.049
nirp3_s1	Student	−1.039	46.000	0.304
	Welch	−1.011	36.867	0.319
	Mann-Whitney	249.000		0.527
nirp3_s2	Student	−0.525	39.000	0.603
	Welch	−0.546	32.910	0.589
	Mann-Whitney	158.500		0.330

Descriptive Statistics

Descriptive Statistics

	p65_s1		p65_s3		casp_s1		mefv_i1_s1		mefv_i2_s1		mefv_i2_s2		mefv_i1.2_s2		nirp3_s1		nirp3_s2	
	Control	FMF	Control	FMF	Control	FMF	Control	FMF	Control	FMF	Control	FMF	Control	FMF	Control	FMF	Control	FMF
Valid	19	25	20	29	20	29	20	28	19	27	20	24	18	29	20	28	15	26
Missing	1	4	0	0	0	0	0	1	1	2	0	5	2	0	0	1	5	3
Mean	−0.312	0.093	−0.453	0.424	−0.436	0.386	−0.810	−0.430	−0.102	−0.452	−0.531	−0.974	−0.340	0.315	−0.163	0.122	−0.182	0.006
Std. Deviation	1.127	1.100	1.290	0.787	1.309	0.705	3.421	2.496	0.786	0.650	1.333	0.578	1.068	0.792	1.023	0.873	1.003	1.158
Minimum	−2.185	−3.511	−2.924	−1.612	−2.856	−1.250	−8.623	−6.513	−1.940	−1.707	−3.009	−1.913	−2.131	−1.296	−3.077	−1.841	−3.439	−4.568
Maximum	1.011	1.224	1.407	1.651	0.965	1.971	2.727	5.207	1.025	0.826	1.588	0.331	1.163	1.934	0.967	1.684	0.712	1.490

Boxplots

p65_s1

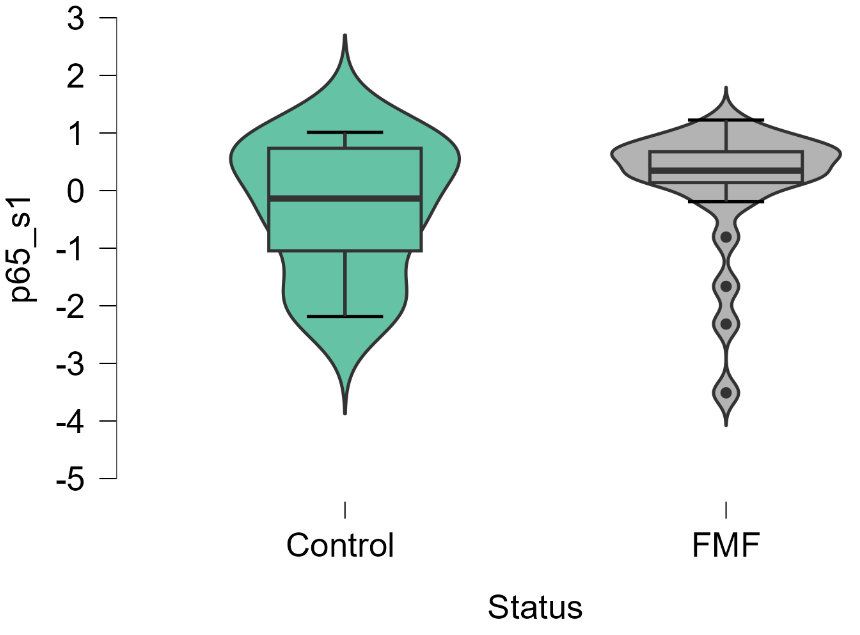

p65_s3

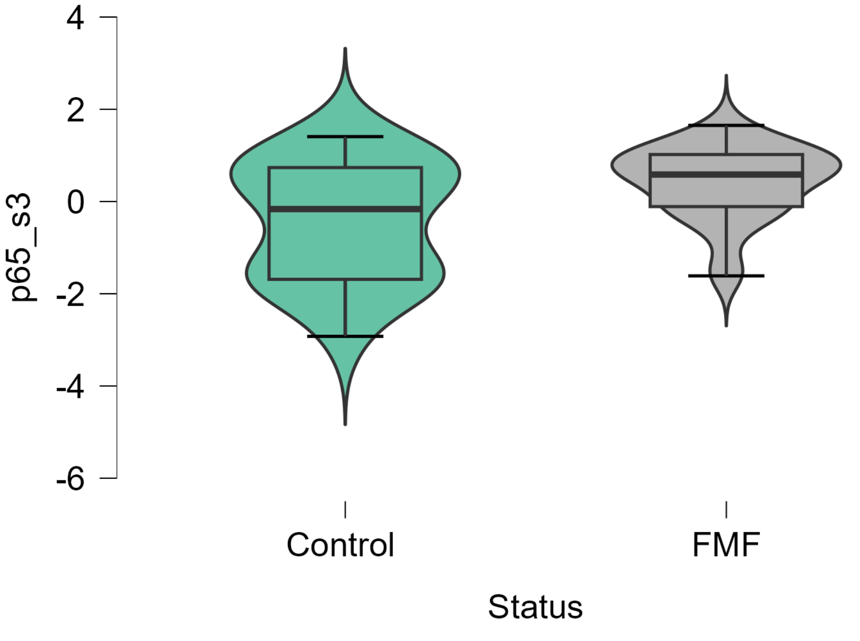

casp_s1

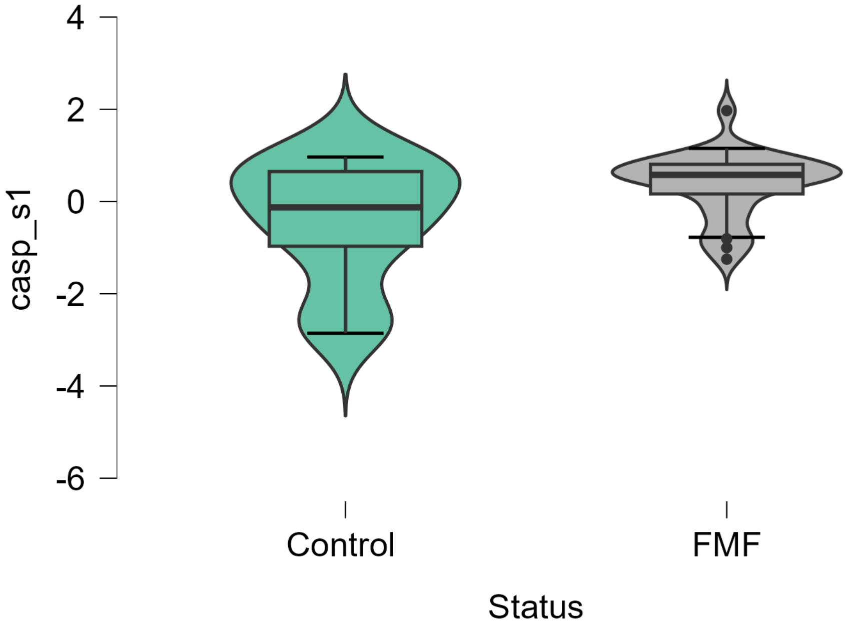

mefv_i1_s1

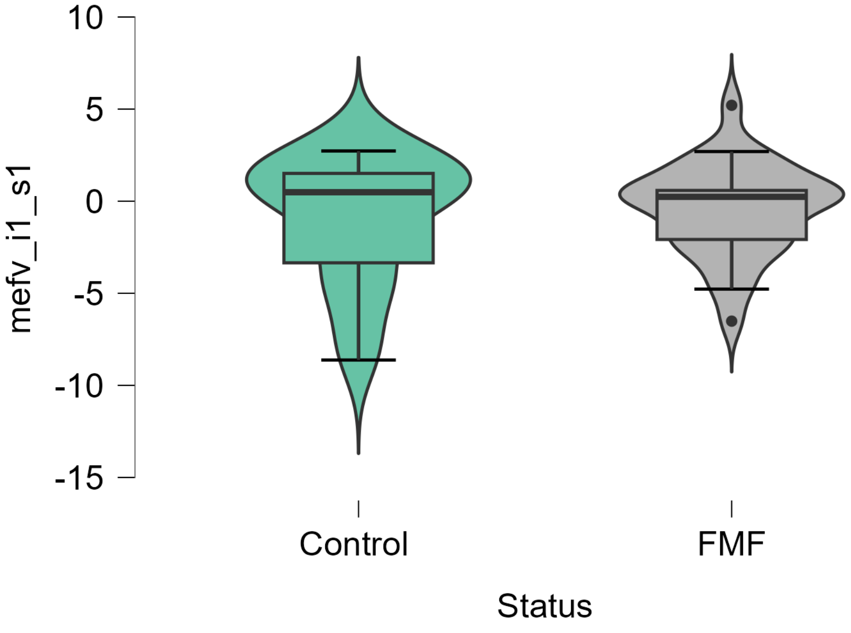

mefv_i2_s1

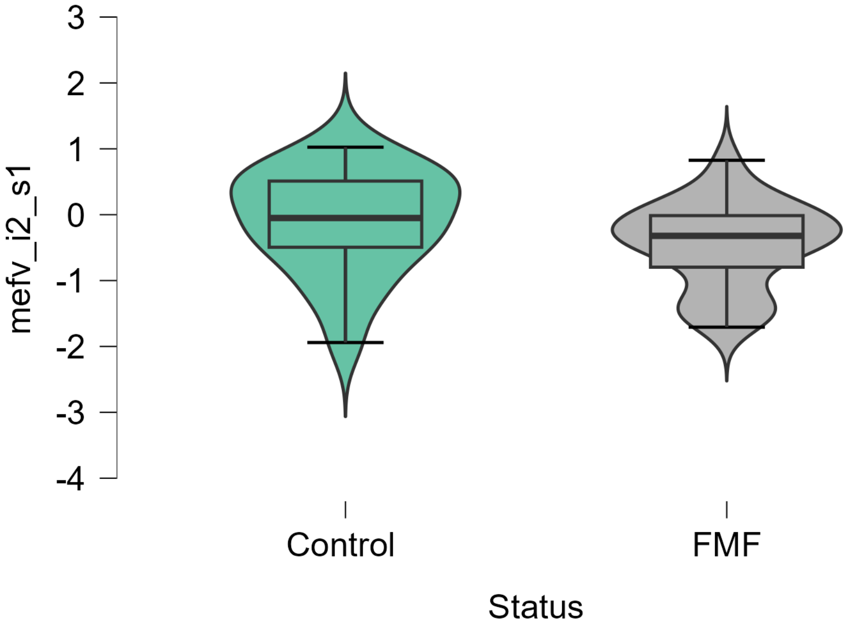

mefv_i2_s2

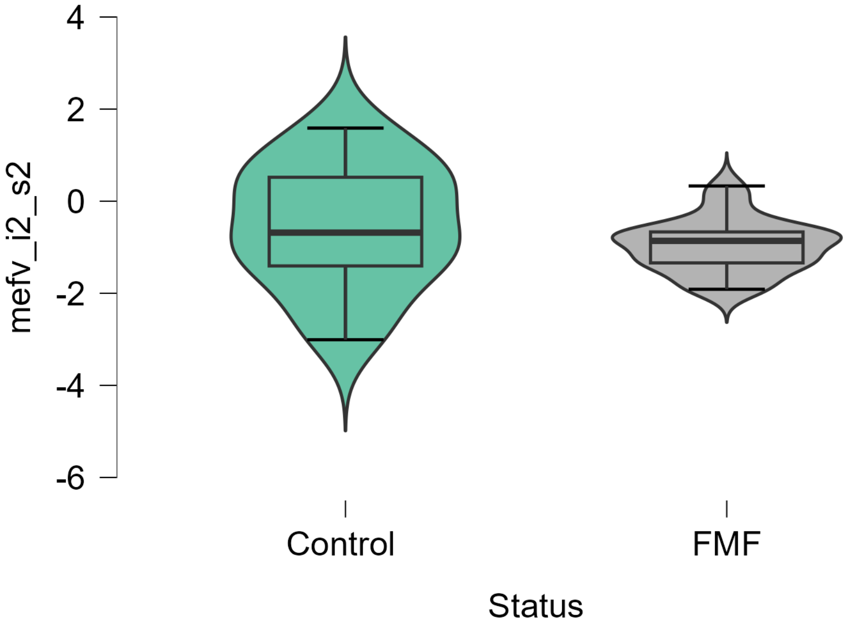

mefv_i1.2_s2

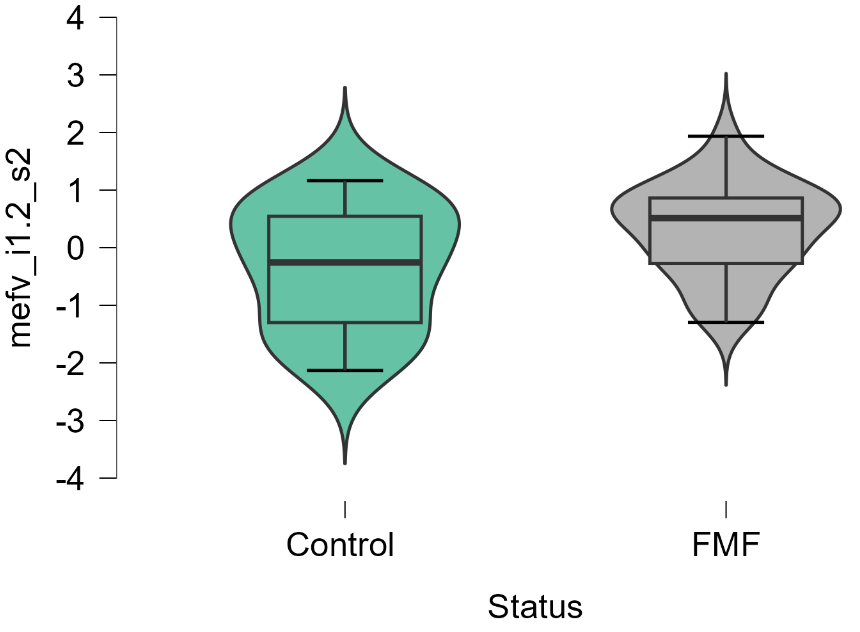

nirp3_s1

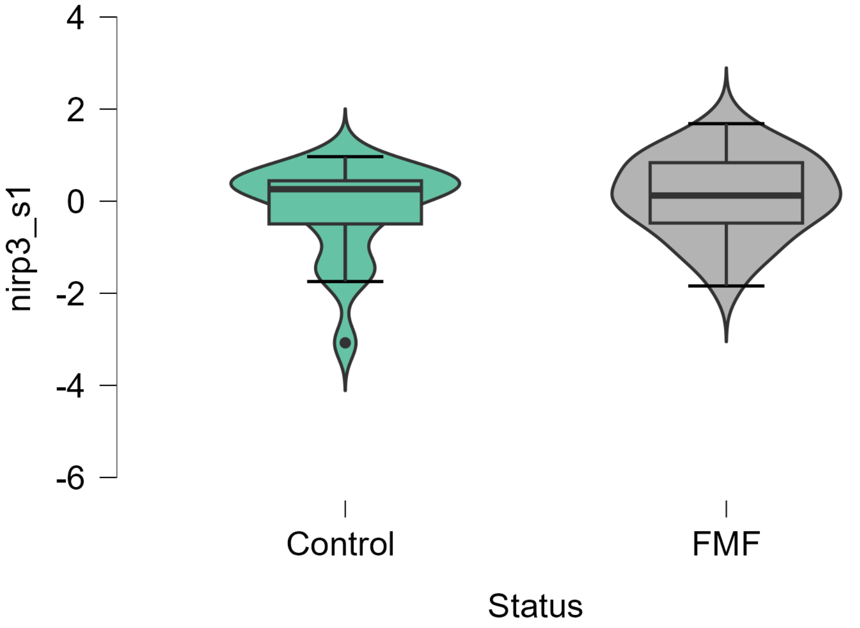

nlrp3_s2

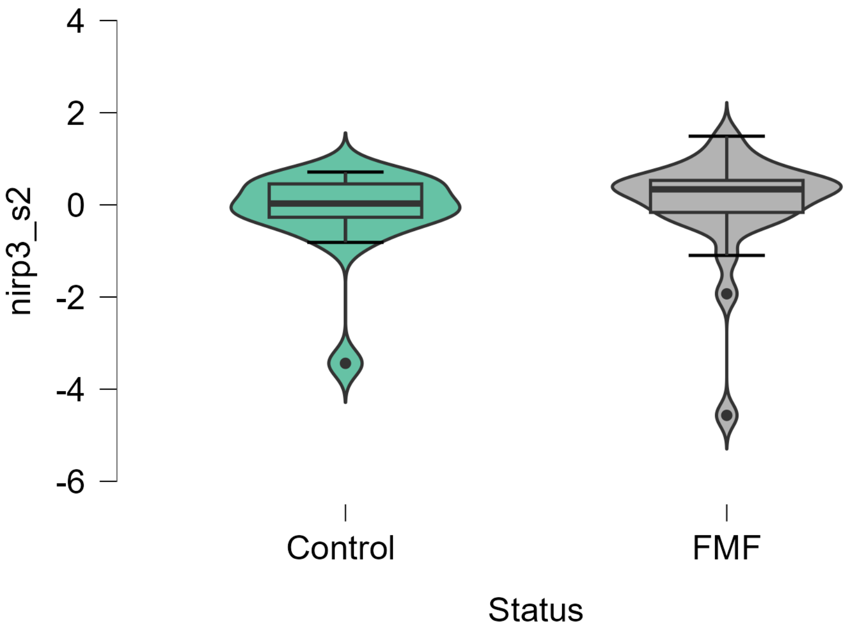

Supplement: Supplementary file 1 [file ijms-25-12958-s001.zip › ijms-3262116-supplementary.pdf]
